# SIRT1 Regulates Mitochondrial Damage in N2a Cells Treated with the Prion Protein Fragment 106–126 via PGC-1α-TFAM-Mediated Mitochondrial Biogenesis

**DOI:** 10.3390/ijms25179707

**Published:** 2024-09-07

**Authors:** Mengyang Zhao, Jie Li, Zhiping Li, Dongming Yang, Dongdong Wang, Zhixin Sun, Pei Wen, Fengting Gou, Yuexin Dai, Yilan Ji, Wen Li, Deming Zhao, Lifeng Yang

**Affiliations:** National Key Laboratory of Veterinary Public Health and Safety, Key Laboratory of Animal Epidemiology of the Ministry of Agriculture and Rural Affairs, National Animal Transmissible Spongiform Encephalopathy Laboratory, College of Veterinary Medicine, China Agricultural University, Beijing 100193, China; bs20213050465@cau.edu.cn (M.Z.);

**Keywords:** SIRT1, mitochondrial quality control, mitochondrial biogenesis, resveratrol, prion disease

## Abstract

Mitochondrial damage is an early and key marker of neuronal damage in prion diseases. As a process involved in mitochondrial quality control, mitochondrial biogenesis regulates mitochondrial homeostasis in neurons and promotes neuron health by increasing the number of effective mitochondria in the cytoplasm. Sirtuin 1 (SIRT1) is a NAD+-dependent deacetylase that regulates neuronal mitochondrial biogenesis and quality control in neurodegenerative diseases via deacetylation of a variety of substrates. In a cellular model of prion diseases, we found that both SIRT1 protein levels and deacetylase activity decreased, and SIRT1 overexpression and activation significantly ameliorated mitochondrial morphological damage and dysfunction caused by the neurotoxic peptide PrP^106–126^. Moreover, we found that mitochondrial biogenesis was impaired, and SIRT1 overexpression and activation alleviated PrP^106–126^-induced impairment of mitochondrial biogenesis in N2a cells. Further studies in PrP^106–126^-treated N2a cells revealed that SIRT1 regulates mitochondrial biogenesis through the PGC-1α-TFAM pathway. Finally, we showed that resveratrol resolved PrP^106–126^-induced mitochondrial dysfunction and cell apoptosis by promoting mitochondrial biogenesis through activation of the SIRT1-dependent PGC-1α/TFAM signaling pathway in N2a cells. Taken together, our findings further describe SIRT1 regulation of mitochondrial biogenesis and improve our understanding of mitochondria-related pathogenesis in prion diseases. Our findings support further investigation of SIRT1 as a potential target for therapeutic intervention of prion diseases.

## 1. Introduction

Prion diseases are a diverse group of chronic, progressive, and fatal transmissible neurodegenerative disorders (NDDs) that affect humans, as well as many animal species. Prion diseases are caused by misfolding of the cellular prion protein (PrP^C^) and accumulation of its disease-associated scrapie prion isoform (PrP^Sc^). The hallmark histopathological features of prion diseases are widespread spongiform encephalopathy, neuronal loss, gliosis, and deposits of variably sized aggregated prion protein in the central nervous system (CNS) [1]. Prions pose a threat to both animals and humans due to their potential to produce fatal diseases that can be transmitted across species barriers and their capacity to mutate, adapt, and evolve, as well as their persistence and extreme resistance to decontamination [2]. At present, no effective therapies exist for human and animal prion diseases. Thus, studies on the pathogenic mechanisms of prion diseases are highly warranted.

PrP^106–126^ is a peptide homologous to residues 106–126 of the human prion protein which sequence is based on the PrP amyloid protein of Gerstmann-Sträussler-Scheinker syndrome patients [3]. The PrP^106–126^ sequence is highly conserved across different species and represents the region considered to be the most critical for initiating the conformational change of PrP^C^ and the transition from PrP^C^ to PrP^Sc^ [4,5]. PrP^106–126^ recapitulates the structural characteristics and biological activities of PrP^Sc^ [6]. The common structural features of PrP^106–126^ and PrP^Sc^ include (1) a β-sheet structure, (2) the formation of toxic soluble oligomers, (3) high fibrillogenic propensity [7], and (4) resistance to proteinase K digestion. PrP^106–126^ also exhibits several biological consequences shared with PrP^Sc^, including (1) neurotoxicity [8], (2) induction of apoptosis, (3) gliosis, (4) autophagy [9], (5) ion channel formation, (6) membrane microviscosity, and (7) oxidative stress. Thus, PrP^106–126^ is commonly used as a model peptide of PrP^Sc^ in the study of prion diseases [10,11,12,13]. N2a cells, derived from mouse neuroblastoma, have been extensively utilized as one of the common in vitro models for the study of prion diseases. Previous research has demonstrated that neuroblastoma cells treated with PrP^106–126^ exhibit prion-related biological consequences, including apoptosis, autophagy, mitophagy, and mitochondrial fission and fusion, among others [14,15,16,17,18]. Building on these findings, the present study utilized PrP^106–126^-treated N2a cells as an in vitro model to investigate potential pathogenic mechanisms of prion disease, particularly those associated with mitochondrial dysfunction.

Recent studies have established that mitochondrial impairment and dysfunction are critical in the pathogenesis of several NDDs, including prion diseases [19,20,21,22]. Mitochondrial quality control (MQC) mechanisms effectively surveil and eliminate damaged mitochondria to restore mitochondrial homeostasis and maintain a healthy pool of mitochondria in cells [23,24,25]. Many studies have shown that mitochondrial biogenesis, as one of the processes involved in MQC, can regulate mitochondrial homeostasis in neurons and promotes neuron health by increasing the number of effective mitochondria in the cytoplasm [20,26,27,28]. Peroxisome proliferator activated receptor γ (PPARγ) coactivator-1α (PGC-1α) and mitochondrial transcription factor A (TFAM) are two critical regulators of mitochondrial biogenesis. PGC-1α activation promotes transcriptional activity to regulate the synthesis of a series of nuclear transcription factors, including TFAM, which is a nuclear genome-encoded transcription factor essential for mitochondrial DNA (mtDNA) replication and transcription [26].

Sirtuin 1 (SIRT1) is a member of the sirtuin family, a group of evolutionarily conserved NAD+-dependent deacetylases [29,30]. SIRT1 regulates various physiological functions, including circadian rhythm and aging, while it is also involved in mitochondrial dysfunction, neuroinflammation, and oxidative stress response through deacetylation of a variety of substrates, including PGC-1α [31,32]. Several studies have shown that SIRT1 participates in the regulation of neuronal MQC mechanisms, particularly mitochondrial biogenesis, in NDDs [33,34,35]. In previous studies, toxic prion protein-exposed neuronal cells exhibited NAD+ depletion [36]. The level of SIRT1 was significantly decreased in scrapie-infected models, and SIRT1 displayed the ability to protect neurons against cell death induced by PrP^106–126^ [37,38]. Moreover, neuronal mitochondrial biogenesis is impaired in NDDs such as Alzheimer’s disease (AD) and Parkinson’s disease (PD), and SIRT1 plays a central role in mitochondrial biogenesis regulation in these diseases [33,34,39].

Despite our understanding of the above, whether mitochondrial biogenesis is impaired in prion diseases and whether SIRT1 regulates mitochondrial biogenesis through the PGC-1α-TFAM pathway have not been fully studied. Thus, in the present study, we aimed to investigate the functional role of, and regulatory mechanisms enacted by, SIRT1 in mitochondrial biogenesis during prion disease. Our studies ultimately demonstrate that SIRT1 plays an important role in the regulation of mitochondrial biogenesis through the PGC-1α-TFAM pathway, and SIRT1 may represent a potential target for therapeutic intervention in prion diseases. Additionally, resveratrol emerges as a promising candidate for prion disease treatment, with its pharmacological effects primarily mediated via the SIRT1-PGC-1α-TFAM pathway.

## 2. Results

### 2.1. SIRT1 Expression and Deacetylase Activity Are Downregulated in PrP^106–126^-Exposed N2a Cells

SIRT1 is a NAD+-dependent deacetylase involved in the regulation of neuronal MQC mechanisms in aging and NDDs, including AD and PD [29,30,33,34]. Previous studies demonstrated that PrP^106–126^-exposed neuronal cells exhibit NAD+ depletion and that SIRT1 regulates PrP^106–126^-induced neuronal cell death [37,40].

**Figure 1:**
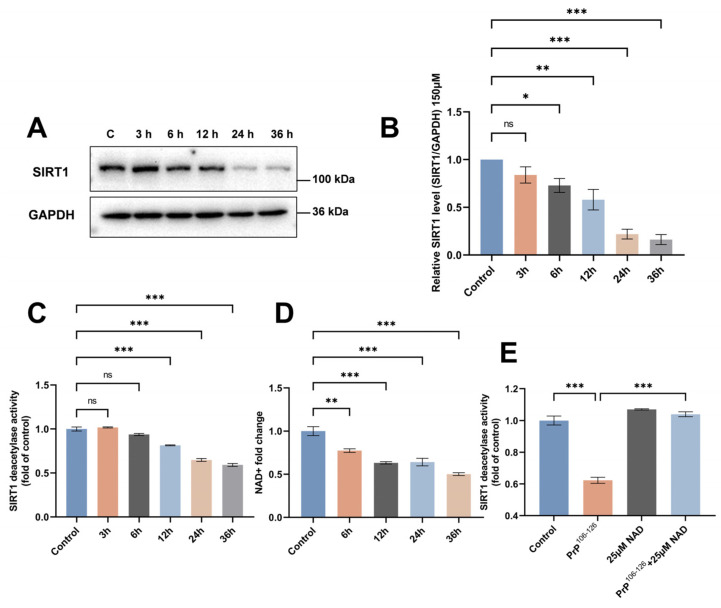
SIRT1 expression and deacetylase activity are downregulated in PrP^106–126^-exposed N2a cells. (**A**) Western blots of SIRT1 in N2a cells exposed to 150 μM PrP^106–126^. (**B**) Quantitation of SIRT1 expression levels shown in (**A**). (**C**) Relative SIRT1 deacetylase activity in PrP^106–126^-exposed N2a cells, measured using a SIRT1 assay kit. (**D**) Relative intracellular NAD+ levels. (**E**) Relative SIRT1 deacetylase activity measured after 25 μM NAD+ supplementation in PrP^106–126^-exposed N2a cells, measured using a SIRT1 assay kit. Data are expressed as the mean ± SEM. Statistical significance was analyzed via ordinary one-way ANOVA with Tukey’s multiple comparisons test for (**B**–**E**). *n* = at least 3 biologically independent treatments of cells for each. ns, not significant; * *p* < 0.05; ** *p* < 0.01; *** *p* < 0.001.

To determine whether SIRT1 participates in the regulation of neuronal MQC in prion diseases, we first measured the expression of SIRT1 in PrP^106–126^-treated N2a cells. N2a cells were cultured with PrP^106–126^ over a time course (3, 6, 12, 24, and 36 h) and concentration gradient (100 μM, 150 μM, and 200 μM). We found that expression of SIRT1 began to decrease after incubation with 150 μM PrP^106–126^ for 6 h and decreased significantly by 24 h and 36 h (Figure 1A,B). SIRT1 expression declined in N2a cells treated with 100 μM PrP^106–126^ for 24 or 36 h (Figure S1A,B). In the 200 μM PrP^106–126^ treatment group, SIRT1 was decreased at the first time point we tested (3 h; Figure S1C,D). Based on the data obtained, the lowest concentration and shortest duration that elicited a significant change in SIRT1 expression were selected for the subsequent experiments in this study. Specifically, 150 μM PrP^106–126^ was administered to N2a cells for a duration of 24 h in the following experiments. We also examined SIRT1 deacetylase activity in N2a cells using a SIRT1 assay kit. SIRT1 deacetylase activity decreased over time following exposure to PrP^106–126^, with a significant decrease in activity observed starting 12 h after PrP^106–126^ treatment (Figure 1C).

SIRT1 activity is influenced by the intracellular NAD+ levels. Next, we evaluated changes in intracellular NAD+ following PrP^106–126^ exposure. The NAD+ levels decreased over time following PrP^106–126^ treatment, which was consistent with the observed change in SIRT1 deacetylase activity in PrP^106–126^-treated N2a cells (Figure 1D). We found that supplementation with NAD+ during PrP^106–126^ treatment was able to restore SIRT1 deacetylase activity to that observed under normal conditions (Figure 1E). Finally, our study revealed that, at the selected time point (24 h) or concentration (150 μM), cell viability was influenced by another variable. Specifically, when N2a cells were exposed to 150 μM PrP^106–126^ for different durations, or when treated with varying concentrations of PrP^106–126^ for 24 h, cell viability decreased as the treatment duration or concentration of PrP^106–126^ increased, respectively (Figure S1E,F). Lastly, it should be noted that, compared to PrP^106–126^-exposed cells, the cell viability of scramble PrP^106–126^-exposed N2a cells did not decrease (Figure S1E), indicating that the observed effects were specifically due to PrP^106–126^.

The above results demonstrate that both the SIRT1 protein levels and deacetylase activity decreased in N2a cells after 24 h of PrP^106–126^ treatment and that SIRT1 deacetylase activity is related to the level of NAD+ in PrP^106–126^-exposed N2a cells.

### 2.2. SIRT1 Participates in PrP^106–126^-Induced Mitochondrial Morphological Damage and Dysfunction

**Figure 2:**
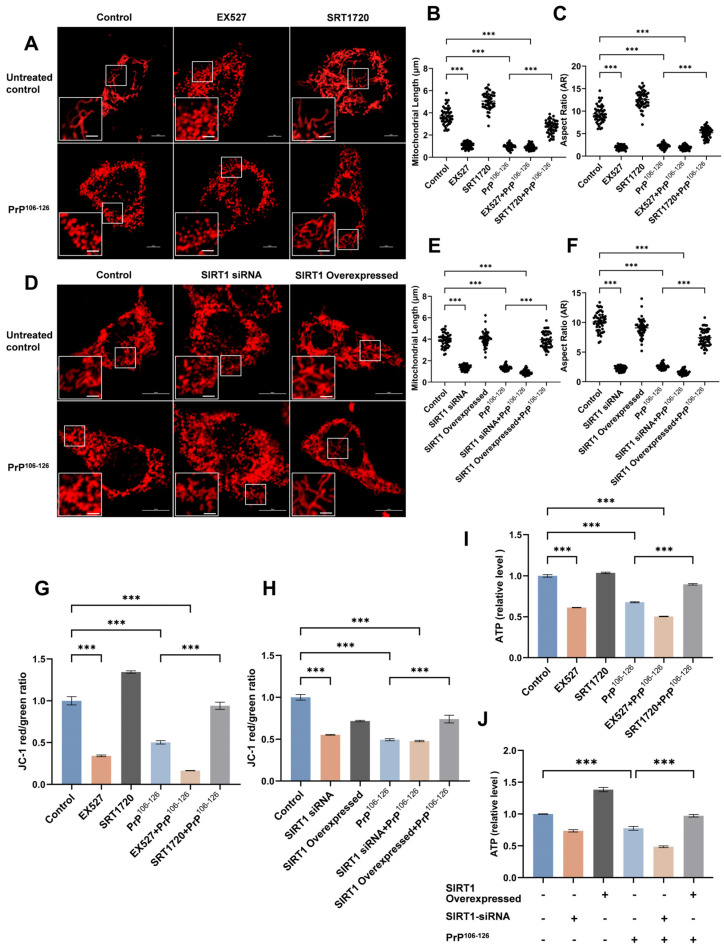
SIRT1 regulates mitochondrial morphological damage and dysfunction in PrP^106–126^-exposed N2a cells. (**A**) Immunofluorescence images of DsRed-Mito-tagged mitochondria showing differing morphologies following EX527, SRT1720, and PrP^106–126^ treatments. Scale bar: zoom out = 5 μm, scale bar: zoom in = 2 μm. (**B**) Mitochondrial lengths of N2a cells (*n* = 50) in (**A**). (**C**) Mitochondrial aspect ratios of N2a cells (*n* = 50) in (**A**). (**D**) Immunofluorescence images of DsRed-Mito-tagged mitochondria showing differing morphologies after SIRT1 knockdown, overexpression, and PrP^106–126^ treatment. Scale bar: zoom out = 10 μm, scale bar: zoom in = 2 μm. (**E**) Mitochondrial lengths of N2a cells (*n* = 50) in (**D**). (**F**) Mitochondrial aspect ratios of N2a cells (*n* = 50) in (**D**). (**G**) JC-1 was used to quantify changes in the mitochondrial membrane potential (MMP) in EX527-, SRT1720-, and PrP^106–126^-treated N2a cells. Changes in MMP indicate damage to the mitochondria. (**H**) JC-1 was used to quantify changes in MMP in N2a cells after SIRT1 knockdown, overexpression, and PrP^106–126^ treatment. (**I**) Relative intracellular ATP levels in EX527-, SRT1720-, and PrP^106–126^-treated N2a cells. (**J**) Relative intracellular ATP levels in EX527-, SRT1720-, and PrP^106–126^-treated N2a cells. Data are expressed as the mean ± SEM. Statistical significance was analyzed via ordinary one-way ANOVA with Tukey’s multiple comparisons test for (**B**,**C**,**E**,**F**,**G**,**J**). *n* = at least 3 biologically independent treatments/transfections of cells for each. *** *p* < 0.001.

In our previous studies, we observed mitochondrial morphological damage and dysfunction in prion disease models [17,18,41]. To explore whether mitochondrial damage is associated with the changes in SIRT1 expression and deacetylase activity observed in prion diseases, we overexpressed or knocked down SIRT1 (using treatment with *SIRT1* siRNA) in N2a cells. The expression of SIRT1 protein decreased by approximately 40% following siRNA transfection and increased by approximately 35% upon SIRT1 overexpression (Figure 2 in below). SRT1720 was developed as a potent and specific agonist of SIRT1, binding to the SIRT1 enzyme-peptide substrate complex. It has been demonstrated to enhance the deacetylation of SIRT1 target proteins in both cellular and animal model systems. Conversely, EX527 inhibits SIRT1 activity by competing for SIRT1 NAD+ binding sites and has been widely used as a selective SIRT1 inhibitor. In our study, we modulated the activity of SIRT1 in N2a cells by supplementing with SRT1720 or EX527, respectively [42,43,44].

The results revealed that activation (Figure 2A–C) and overexpression (Figure 2D–F) of SIRT1 were able to rescue the mitochondrial fragmentation induced by PrP^106–126^. Additionally, the mitochondrial membrane potential (MMP) (Figure 2G,H) and ATP levels (Figure 2I,J) were decreased in PrP^106–126^-exposed N2a cells and were subsequently increased following SIRT1 overexpression or activation. SIRT1 knockdown and inhibition intensified the fragmentation of mitochondria (Figure 2A–F) and reduced the MMP (Figure 2G,H) and intracellular ATP levels (Figure 2I,J) in N2a cells.

Taken together, these results suggest that SIRT1 is involved in PrP^106–126^-induced mitochondrial morphological damage and dysfunction. SIRT1 overexpression and activation significantly ameliorate mitochondrial impairment following PrP^106–126^ treatment.

### 2.3. SIRT1 Overexpression and Activation Alleviate PrP^106–126^-Induced Impairment of Mitochondrial Biogenesis

As one of the processes involved in MQC, mitochondrial biogenesis can increase the number of functional mitochondria in the cytoplasm and thus regulate neuronal mitochondrial homeostasis and promote neuron health in the setting of NDD [20,27,45]. Our previous studies demonstrated that the mtDNA copy numbers decreased in prion-infected C57 mice brains and in primary neurons incubated with PrP^106–126^ [18]. Based on the above results and previous studies, we speculated that mitochondrial biogenesis may be impaired and that SIRT1 may be involved in the regulation of mitochondrial biogenesis in prion diseases.

To test our hypothesis, we evaluated mitochondrial biogenesis and the role of SIRT1 in this process in N2a cells treated with PrP^106–126^. We first activated or inhibited SIRT1 activity, as well as overexpressed or knocked down SIRT1 in N2a cells, prior to PrP^106–126^ incubation. Mitochondrial biogenesis entails the replication of mitochondrial DNA (mtDNA) and transcription and translation of mtDNA-encoded genes, as well as loading of phospholipids and nuclear genome-encoded proteins in different mitochondrial subcompartments [46]. Based on this, the following measures, which together reflect mitochondrial biogenesis to some extent, were assessed in N2a cells: (1) mtDNA copy number using real-time PCR, (2) levels of mRNAs for mitochondrial-encoded proteins using real-time PCR, (3) levels of mitochondrial-encoded proteins using Western blot, and (4) levels of nuclear genome-encoded subunit proteins of mitochondrial complexes.

**Figure 3:**
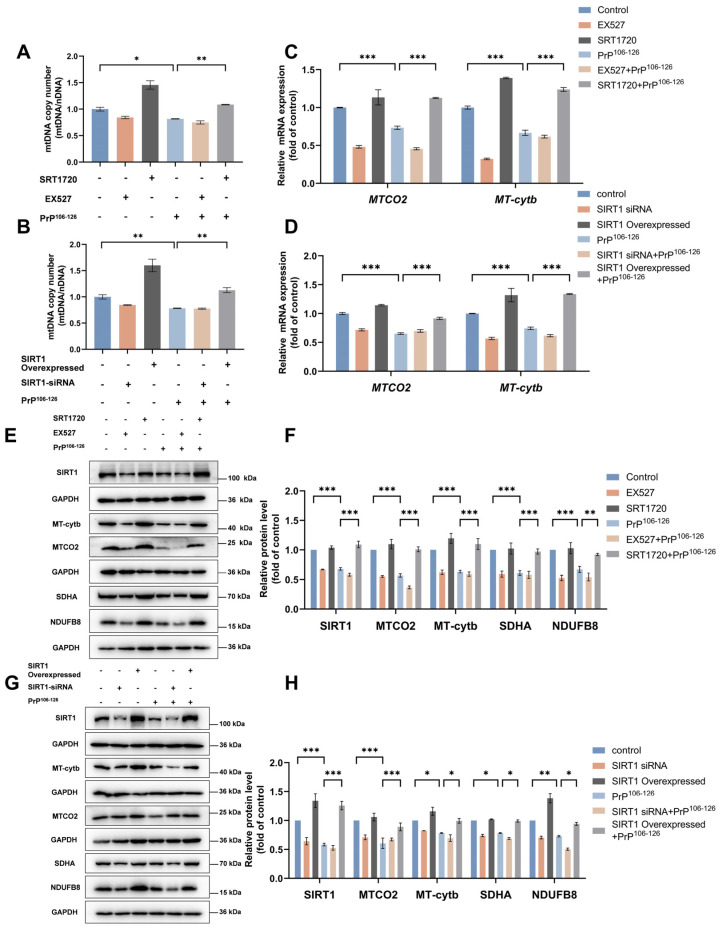
SIRT1 is involved in the regulation of mitochondrial biogenesis impairment caused by PrP^106–126^ treatment. (**A**) Relative mitochondrial DNA copy number (mtDNA/nDNA) in EX527- and SRT1720-treated N2a cells incubated with PrP^106–126^. (**B**) Relative mitochondrial DNA copy number (mtDNA/nDNA) in *SIRT1* siRNA-transfected and SIRT1-overexpressed N2a cells incubated with PrP^106–126^. (**C**) The mRNA levels of mitochondrial-encoded genes (*MTCO2* and *MT*-*Cytb*) in EX527- and SRT1720-treated N2a cells incubated with PrP^106–126^. Levels were calculated relative to the *GAPDH* mRNA level and were normalized to that measured for the control. (**D**) The mRNA levels of mitochondrial-encoded genes (*MTCO2* and *MT*-*Cytb*) in *SIRT1* siRNA-transfected and SIRT1-overexpressed N2a cells incubated with PrP^106–126^. The levels were calculated relative to the *GAPDH* mRNA level and were normalized to that measured for the control. (**E**) Western blots of SIRT1, mitochondrial-encoded proteins (MTCO2 and MT-Cytb), and nuclear genome-encoded subunits of mitochondrial complexes (SDHA and NDUFB8) in EX527- and SRT1720-treated N2a cells incubated with PrP^106–126^. (**F**) Quantitation of the results shown in (**E**). (**G**) Western blots of SIRT1, mitochondrial-encoded proteins (MTCO2 and MT-Cytb), and nuclear genome-encoded subunits of mitochondrial complexes (SDHA and NDUFB8) in *SIRT1* siRNA-transfected and SIRT1-overexpressed N2a cells incubated with PrP^106–126^. (**H**) Quantitation of the results shown in (**G**). Data are expressed as the mean ± SEM. Statistical significance was analyzed via ordinary one-way ANOVA with Tukey’s multiple comparisons test for (**A**,**B**) or two-way ANOVA with Sidak’s multiple comparisons test for (**C**,**D**,**F**,**H**). *n* = at least 3 biologically independent treatments/transfections of cells for each. * *p* < 0.05; ** *p* < 0.01; *** *p* < 0.001.

Our results showed that PrP^106–126^-treated N2a cells contain approximately 70–80% of the mtDNA copy numbers found in the untreated control cells (Figure 3A,B). We also observed that both the levels of mRNA (Figure 3C,D) and protein (Figure 3E–H) of MT-Cytb and MTCO2, two mitochondrial-encoded proteins, were significantly downregulated in N2a cells after PrP^106–126^ incubation. In addition, the levels of NDUFB8 and SDHA, which are the subunits of mitochondrial complexes I and II, respectively, were also downregulated in N2a cells after PrP^106–126^ incubation (Figure 3E–H). We further investigated whether overexpression or activation of SIRT1 could alleviate the mtDNA loss and reduction in mitochondrial proteins caused by PrP^106–126^. Indeed, SIRT1 overexpression or activation blocked the PrP^106–126^-induced loss of mtDNA and reduction of mitochondrial-encoded proteins and nuclear genome-encoded subunits of mitochondrial complexes. The mtDNA/nDNA ratio was restored to preincubation levels in SIRT1-overexpressed or SIRT1-activated N2a cells that were incubated with PrP^106–126^ compared to untreated control N2a cells (Figure 3A,B). The mRNA (Figure 3C,D) and protein levels (Figure 3E–H) for MT-Cytb and MTCO2 were similar or even slightly greater in cells overexpressing or activating SIRT1 treated with PrP^106–126^ when compared to untreated control cells, as were the levels of NDUFB8 and SDHA (Figure 3E–H).

In summary, these results indicate that mitochondrial biogenesis is impaired, and SIRT1 is a crucial regulator of mitochondrial biogenesis in PrP^106–126^-treated N2a cells. SIRT1 overexpression and activation alleviates PrP^106–126^-induced impairment of mitochondrial biogenesis.

### 2.4. TFAM Is Required for SIRT1-Mediated Mitochondrial Biogenesis in PrP^106–126^-Treated N2a Cells

We next examined the mechanisms by which SIRT1 regulates mitochondrial biogenesis. Several studies have demonstrated that TFAM plays an essential role in mtDNA stability and is necessary for the transcription and replication of mtDNA, which are key processes in mitochondrial biogenesis [47,48].

**Figure 4:**
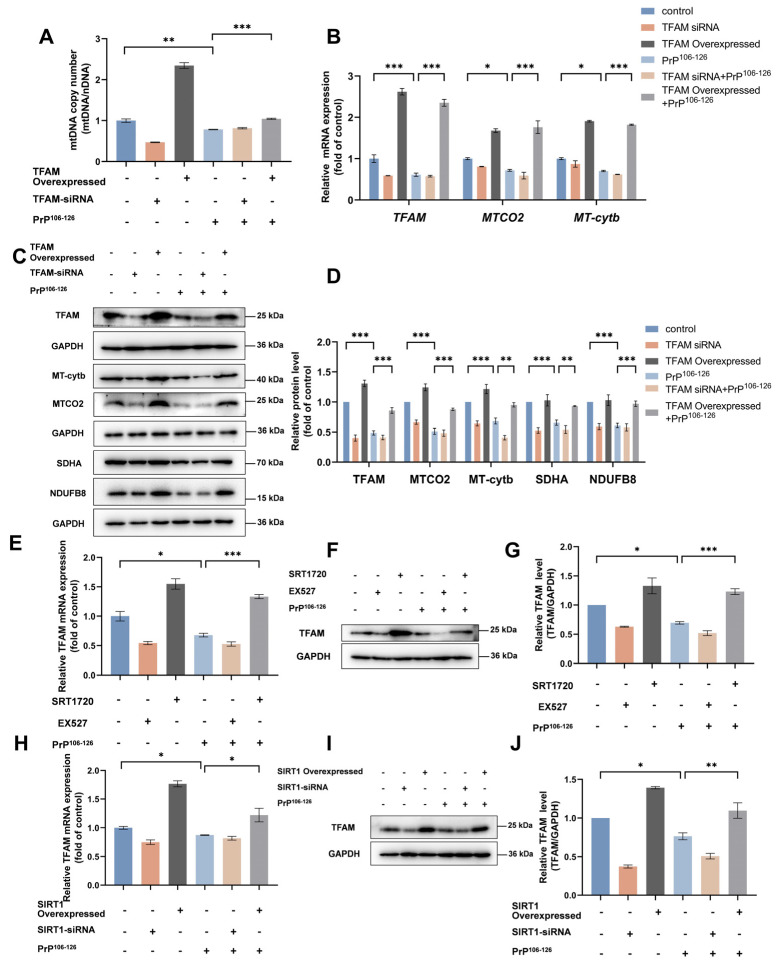
TFAM is required for SIRT1-mediated mitochondrial biogenesis in PrP^106–126^-treated N2a cells. (**A**) Relative mitochondrial DNA copy numbers (mtDNA/nDNA) in TFAM siRNA-transfected and TFAM-overexpressed N2a cells incubated with PrP^106–126^. (**B**) The mRNA levels of mitochondrial-encoded genes (*MTCO2* and *MT*-*Cytb*) in *TFAM* siRNA-transfected and TFAM-overexpressed N2a cells incubated with PrP^106–126^. (**C**) Western blots of TFAM, mitochondrial-encoded proteins (MTCO2 and MT-Cytb), and nuclear genome-encoded subunits of mitochondrial complexes (SDHA and NDUFB8) in *TFAM* siRNA-transfected and TFAM-overexpressed N2a cells incubated with PrP^106–126^. (**D**) Quantitation of the results shown in (**C**). (**E**) *TFAM* mRNA levels in EX527- and SRT1720-treated N2a cells incubated with PrP^106–126^. (**F**) Western blots showing TFAM levels in EX527- and SRT1720-treated N2a cells incubated with PrP^106–126^. (**G**) Quantitation of the results shown in (**F**). (**H**) *TFAM* mRNA levels in *SIRT1* siRNA-transfected and SIRT1-overexpressed N2a cells incubated with PrP^106–126^. (**I**) Western blots showing the TFAM levels in *SIRT1* siRNA-transfected and SIRT1-overexpressed N2a cells incubated with PrP^106–126^. (**J**) Quantitation of the results shown in (**I**). For all mRNA measurements, the levels were calculated relative to the *GAPDH* mRNA level and were normalized to that measured for the control. Data are expressed as the mean ± SEM. Statistical significance was analyzed via ordinary one-way ANOVA with Tukey’s multiple comparisons test for (**A**,**E**,**G**,**H**,**J**) or two-way ANOVA with Sidak’s multiple comparisons test for (**B**,**D**). *n* = at least 3 biologically independent treatments/transfections of cells for each. * *p* < 0.05; ** *p* < 0.01; *** *p* < 0.001.

To validate the role of TFAM in mitochondrial biogenesis, we generated TFAM-overexpressed, as well as TFAM-knockdown, N2a cells (cells treated with *TFAM* siRNA). We observed that TFAM positively regulates mtDNA copy numbers (Figure 4A), as well the expression of mitochondrial-encoded proteins and nuclear genome-encoded subunits of mitochondrial complexes (Figure 4B–D) in PrP^106–126^-treated N2a cells, suggesting that TFAM participates in the regulation of mitochondrial biogenesis.

To further analyze whether TFAM participates in SIRT1-mediated mitochondrial biogenesis, we measured the levels of *TFAM* mRNA and protein in N2a cells. In contrast to untreated cells, SIRT1-knockdown or SIRT1-inhibited cells and PrP^106–126^-treated cells showed a decrease in both *TFAM* mRNA (Figure 4E,H) and TFAM protein (Figure 4F,G,I,J). The reduced TFAM levels caused by PrP^106–126^ were rescued by SIRT overexpression or activation in N2a cells.

Collectively, our results indicate that TFAM is an important regulator of mitochondrial biogenesis and is required for SIRT1-mediated signaling in our model.

### 2.5. SIRT1 Regulates Mitochondrial Biogenesis through the PGC-1α-TFAM Signaling Pathway in a PrP^106–126^-Induced Prion Model

PGC-1α is a direct substrate of SIRT1; deacetylation of PGC-1α by SIRT1 stimulates PGC-1α activity. Growing evidence suggests that PGC-1α is a key regulator of mitochondrial biogenesis and that PGC-1α expression is directly correlated with mitochondrial biogenesis activity. The expression of many nuclear genes, including *TFAM*, which plays a crucial role in the replication and transcription of the mitochondrial genome itself, depend on the activation of PGC-1α [49,50].

**Figure 5:**
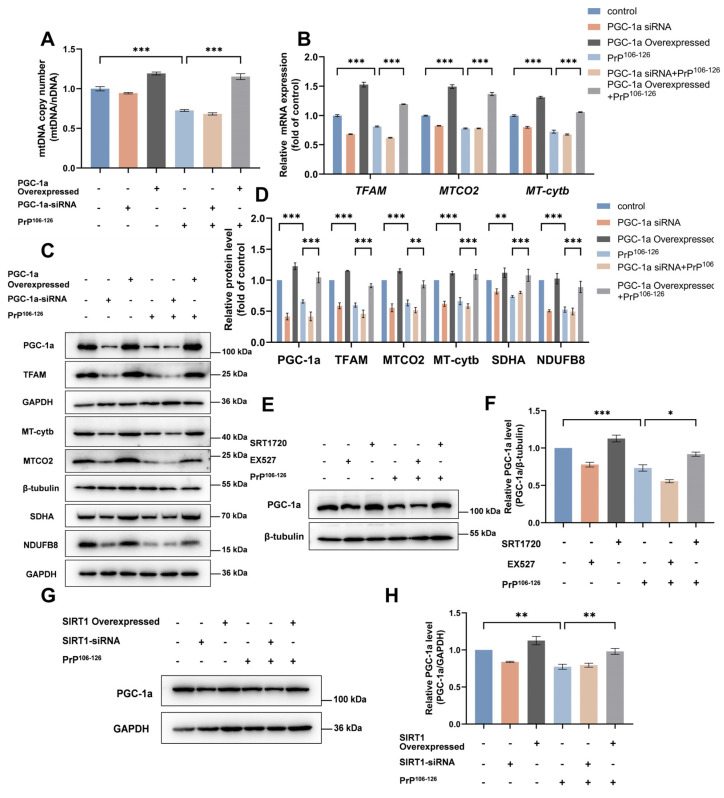
PGC-1α participates in SIRT1-mediated mitochondrial biogenesis in PrP^106–126^-treated N2a cells. (**A**) Relative mitochondrial DNA copy numbers (mtDNA/nDNA) in *PGC-1α* siRNA-transfected and PGC-1α-overexpressed N2a cells incubated with PrP^106–126^. (**B**) The mRNA levels for *TFAM* and mitochondrial-encoded genes (*MTCO2* and *MT*-*Cytb*) in *PGC-1α* siRNA-transfected and PGC-1α-overexpressed N2a cells incubated with PrP^106–126^. (**C**) Western blots of PGC-1α, TFAM, mitochondrial-encoded proteins (MTCO2 and MT-Cytb), and nuclear genome-encoded subunits of mitochondrial complexes (SDHA and NDUFB8) in *PGC-1α* siRNA-transfected and PGC-1α-overexpressed N2a cells incubated with PrP^106–126^. (**D**) Quantitation of the results shown in (**C**). (**E**) Western blots of PGC-1α in EX527- and SRT1720-treated N2a cells incubated with PrP^106–126^. (**F**) Quantitation of the results shown in (**E**). (**G**) Western blot of PGC-1α in *SIRT1* siRNA-transfected and SIRT1-overexpressed N2a cells incubated with PrP^106–126^. (**H**) Quantitation of the results shown in (**G**). For all mRNA measurements, the levels were calculated relative to the *GAPDH* mRNA level and were normalized to that measured for the control. Data are expressed as the mean ± SEM. Statistical significance was analyzed via ordinary one-way ANOVA with Tukey’s multiple comparisons test for (**A**,**F**,**H**) or two-way ANOVA with Sidak’s multiple comparisons test for (**B**,**D**). *n* = at least 3 biologically independent treatments/transfections of cells for each. * *p* < 0.05; ** *p* < 0.01; *** *p* < 0.001.

To probe the role of PGC-1α in mitochondrial biogenesis in the context of prion diseases, we constructed PGC-1α-overexpressed and PGC-1α-knockdown N2a cells. We found that, following treatment with PrP^106–126^, PGC-1α overexpression restored the mRNA (Figure 5B) and protein levels (Figure 5C,D) for TFAM, MTCO2, and MT-cytb to levels comparable to or even greater than those observed for the control in N2a cells. Similar effects were observed for the mtDNA copy number (Figure 5A). and protein levels of SDHA and NDUFB8 (Figure 5C,D). PGC-1α knockdown elicited the opposite effect of PGC-1α overexpression. These results indicated that PGC-1α participates in the regulation of mitochondrial biogenesis via TFAM signaling. We subsequently evaluated the effect of changes in SIRT1 expression and activity on PGC-1α expression. The resulting data revealed that SIRT1 overexpression and activation reversed the decrease in PGC-1α caused by PrP^106–126^ (Figure 5E–H).

**Figure 6:**
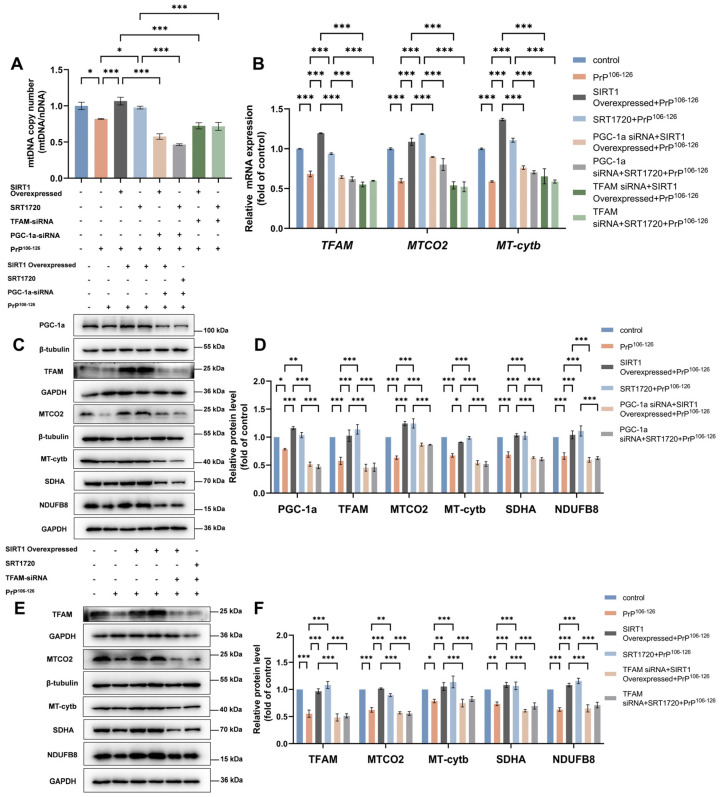
Knockdown of PGC-1α or TFAM negated the beneficial effects of SIRT1 overexpression and activation on mitochondrial biogenesis in PrP^106–126^-treated N2a cells. (**A**) Relative mitochondrial copy numbers (mtDNA/nDNA). (**B**) The relative mRNA levels for *TFAM* and the mitochondrial-encoded genes *MTCO2* and *MT*-*Cytb*. (**C**) Western blots of PGC-1α, TFAM, MTCO2, MT-Cytb, SDHA, and NDUFB8 in *PGC-1α* siRNA-transfected and SIRT1-overexpressed or -activated N2a cells incubated with PrP^106–126^. (**D**) Quantitation of the results shown in (**C**). (**E**) Western blots of TFAM, MTCO2, MT-Cytb, SDHA, and NDUFB8 in *TFAM* siRNA-transfected and SIRT1-overexpressed or -activated N2a cells incubated with PrP^106–126^. (**F**) Quantitation of the results shown in (**E**). The experiments shown in (**A**,**B**) include the following treatment groups: control, PrP^106–126^ incubation, SIRT1-overexpressed and PrP^106–126^ incubation, SRT1720 and PrP^106–126^ co-treatment, SIRT1-overexpressed and PrP^106–126^ incubation after PGC-1α knockdown, SRT1720 and PrP^106–126^ co-treatment after PGC-1α knockdown, SIRT1-overexpressed and PrP^106–126^ incubation after TFAM knockdown, and SRT1720 and PrP^106–126^ co-treatment after TFAM knockdown. All mRNA levels were calculated relative to the *GAPDH* mRNA level and were normalized to that measured for the control. Data are expressed as the mean ± SEM. Statistical significance was analyzed via ordinary one-way ANOVA with Tukey’s multiple comparisons test for (**A**) or two-way ANOVA with Sidak’s multiple comparisons test. for (**B**,**D**,**F**); *n* = at least 3 biologically independent treatments/transfections of cells for each. * *p* < 0.05; ** *p* < 0.01; *** *p* < 0.001.

Next, we further assessed whether the knockdown of either PGC-1α or TFAM can negate the beneficial effects of SIRT1 overexpression or activation on mitochondrial biogenesis. We transfected *TFAM* siRNA or *PGC-1α* siRNA in N2a cells before SIRT1 overexpression or activation. We observed that these beneficial effects of SIRT1 overexpression or activation on restoring PrP^106–126^-induced loss of the mtDNA copy number (Figure 6A) and reduction in the expression of SDHA, NDUFB8, and mitochondrial-encoded proteins (Figure 6B–F) were all inhibited by PGC-1α or TFAM knockdown in N2a cells.

Taken together, the above data demonstrate that SIRT1 regulates mitochondrial biogenesis through the PGC-1α-TFAM signaling pathway in a PrP^106–126^-induced prion disease model.

### 2.6. Resveratrol (RSV) Ameliorates PrP^106–126^-Induced Mitochondrial Dysfunction by Activating SIRT1-Dependent Mitochondrial Biogenesis in N2a Cells

RSV is a naturally occurring polyphenol that exhibits neuroprotective effects in several NDDs. Several studies have shown that RSV regulates mitochondrial biogenesis via multiple mechanisms facilitated by different key effectors (including SIRT1, AMPK, PGC-1α, and cAMP) [51,52,53].

**Figure 7:**
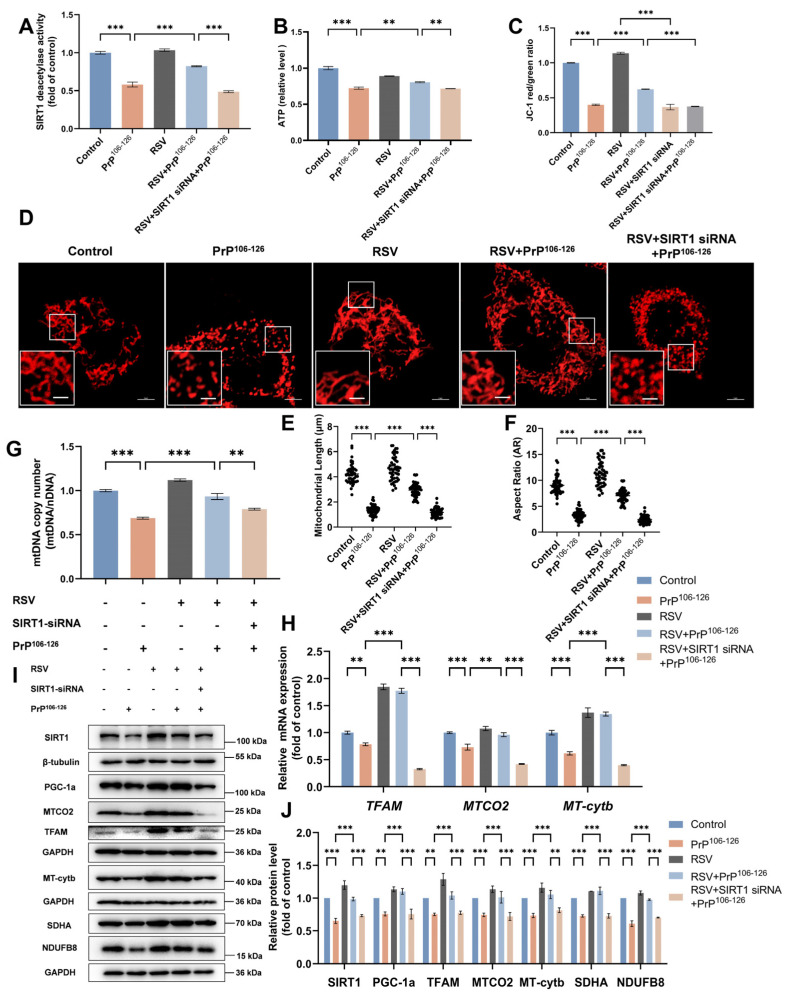
RSV ameliorates PrP^106–126^-induced mitochondrial dysfunction by activating SIRT1-dependent mitochondrial biogenesis in N2a cells. (**A**) Relative SIRT1 deacetylase activity. (**B**) Relative intracellular ATP levels. (**C**) JC-1 was used to quantify changes in the mitochondrial membrane potential (MMP), which indicates damage to the mitochondria. (**D**) Immunofluorescence images of DsRed-Mito-tagged mitochondria showing differing morphologies. Scale bar: zoom out = 5 μm, scale bar: zoom in = 2 μm. (**E**) Mitochondrial length of the N2a cells (*n* = 50) in (**D**). (**F**) Mitochondrial aspect ratio of the N2a cells (*n* = 50) in (**D**). (**G**) Relative mitochondrial copy numbers (mtDNA/nDNA). (**H**) The relative mRNA levels for *TFAM* and the mitochondrial-encoded genes *MTCO2* and *MT*-*Cytb*. (**I**) Western blots of SIRT1, PGC-1α, TFAM, mitochondrial-encoded proteins (MTCO2 and MT-Cytb), and nuclear genome-encoded subunits of mitochondrial complexes (SDHA and NDUFB8). (**J**) Quantitation of the results shown in (**I**). All of the experiments shown in this figure include the following treatment groups: control, PrP^106–126^ incubation, RSV supplement, RSV and PrP^106–126^ co-treatment, and RSV and PrP^106–126^ co-treatment after SIRT1 knockdown. All the mRNA levels were calculated relative to the *GAPDH* mRNA level and were normalized to that measured for the control. Data are expressed as the mean ± SEM. Statistical significance was analyzed via ordinary one-way ANOVA with Tukey’s multiple comparisons test for (**A**–**C**,**E**–**G**) or two-way ANOVA with Sidak’s multiple comparisons test for (**H**,**J**). *n* = at least 3 biologically independent treatments/transfections of cells for each. ns, not significant; ** *p* < 0.01; *** *p* < 0.001.

We observed that pretreatment with RSV caused SIRT1 deacetylase activity to recover from 58% to 82% that in the untreated control group under PrP^106–126^ exposure (Figure 7A). Next, we assessed whether RSV treatment caused changes in mitochondrial morphology and function. RSV pretreatment rescued the mitochondrial fragmentation (Figure 7D–F) and the decrease in the MMP (Figure 7C) and ATP (Figure 7B) levels caused by PrP^106–126^. To explore the mechanism by which RSV tempers PrP^106–126^-induced mitochondrial damage, we again measured metrics related to mitochondrial biogenesis and found that RSV pretreatment reversed the defects in mitochondrial biogenesis caused by PrP^106–126^. Specifically, RSV suppressed the reduction in mtDNA copy numbers (Figure 7G) and changes in the expression of SIRT1, SDHA, NDUFB8, PGC-1α, TFAM, and mitochondrial-encoded proteins at both the transcriptional (Figure 7H) and translational (Figure 7I,J) levels. We also evaluated the effects of SIRT1 on RSV neuroprotection by knocking down SIRT1 prior to treatment with RSV and PrP^106–126^. Importantly, we found that the ability of RSV to rescue the mitochondrial dysfunction caused by PrP^106–126^ insult was significantly inhibited by SIRT1 knockdown, suggesting that RSV protects N2a cells from PrP^106–126^-induced mitochondrial dysfunction by activating mitochondrial biogenesis through the SIRT1-dependent PGC-1α/TFAM signaling pathway.

### 2.7. RSV Supplementation Attenuates PrP^106–126^-Induced N2a Cell Apoptosis

We demonstrated that RSV supplementation protects mitochondrial morphology and function from the deleterious effects of PrP^106–126^. We next assessed the effect of RSV on N2a cell survival.

**Figure 8:**
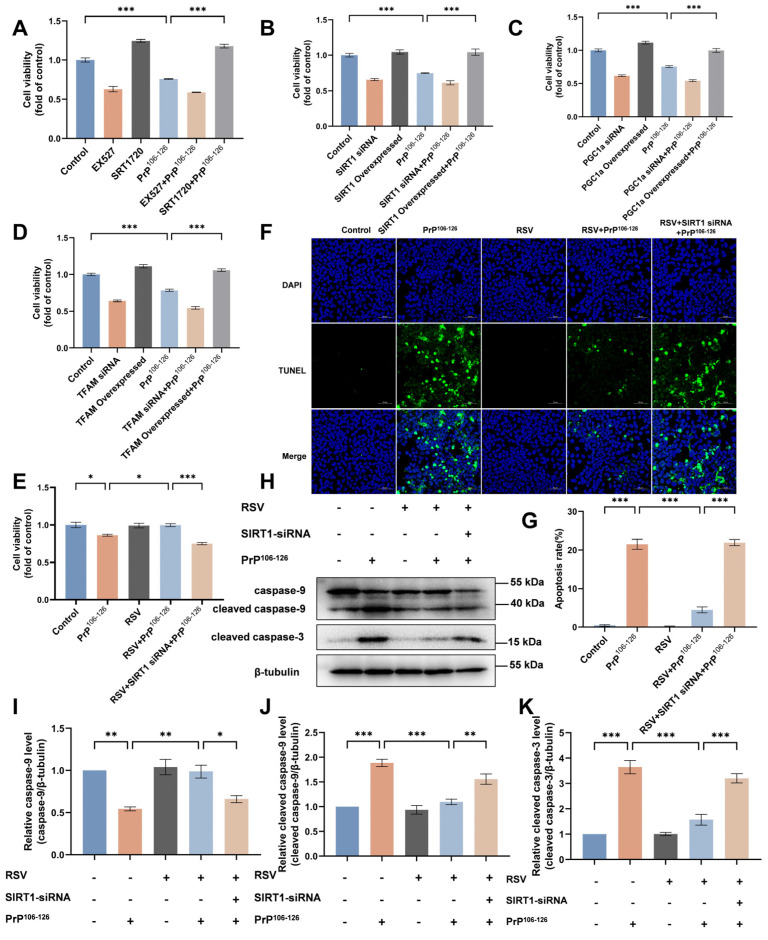
RSV supplementation attenuates PrP^106–126^-induced N2a cell apoptosis. (**A**) Cell viability was assayed using the cell counting CCK-8 kit in EX527-, SRT1720-, and PrP^106–126^-treated N2a cells. (**B**) Cell viability was assayed using the cell counting CCK-8 kit in N2a cells after SIRT1 knockdown, overexpression, and PrP^106–126^ treatment. (**C**) Cell viability was assayed using the cell counting CCK-8 kit in N2a cells after PGC-1α knockdown, overexpression, and PrP^106–126^ treatment. (**D**) Cell viability was assayed using the cell counting CCK-8 kit in N2a cells after TFAM knockdown, overexpression, and PrP^106–126^ treatment. (**E**) Cell viability was assayed using the cell counting CCK-8 kit in control or treated N2a cells. (**F**) Confocal images of TUNEL and DAPI staining in N2a cells. Scale bar: 50 μm. (**G**) Proportion of apoptotic cells as quantified by TUNEL staining. (**H**) Western blots of caspase-9, cleaved caspase-9, and cleaved caspase-3 to identify the rates of apoptosis. (**I**–**K**) Quantitation of the results shown in (**H**). The experiments shown in (**E**–**K**) include the following treatment groups: control, PrP^106–126^ incubation, RSV supplement, RSV and PrP^106–126^ co-treatment, and RSV and PrP^106–126^ co-treatment after SIRT1 knockdown. Data are expressed as the mean ± SEM. Statistical significance was analyzed via ordinary one-way ANOVA with Tukey’s multiple comparisons test for (**A**–**E**,**G**,**I**–**K**). *n* = at least 3 biologically independent treatments/transfections of cells for each. * *p* < 0.05; ** *p* < 0.01; *** *p* < 0.001.

We first examined cell viability using the CCK-8 assay under SIRT1, TFAM, or PGC-1α interventions. We found that the percentage of surviving cells was reduced in PrP^106–126^-incubated N2a cells relative to the untreated control. SIRT1, PGC-1α, or TFAM overexpression and SIRT1 activation restored cell viability to levels similar to those observed for the control (Figure 8A–D).

Next, we found that RSV pretreatment enhanced cell survival and inhibited apoptosis in PrP^106–126^-incubated N2a cells. RSV supplementation restored the reduction of cell viability caused by PrP^106–126^ (Figure 8E). Specifically, following PrP^106–126^ incubation in N2a cells, the proportion of apoptotic cells (Figure 8F,G) and levels of apoptosis-related factors (cleaved caspase-3 and cleaved caspase-9) (Figure 8C,E–G) were significantly increased, while the caspase-9 levels decreased, indicating the activation of N2a cell apoptosis (Figure 8H–K). Notably, RSV supplementation restored the levels of apoptosis-related factors to levels similar to those observed for the control. The protective effect of RSV supplementation, which inhibited apoptosis and protected cells from cell death caused by PrP^106–126^, was limited by SIRT1 knockdown.

Altogether, our results suggest that SIRT1, TFAM, and PGC-1α interventions influence not only mitochondrial biogenesis but also the overall health and viability of PrP^106–126^-induced N2a cells. RSV supplementation attenuates PrP^106–126^-induced N2a cell apoptosis, and SIRT1 plays a critical role in this process.

## 3. Discussion

In this study, we investigated whether impaired mitochondrial biogenesis, induced in N2a cells by the prion peptide PrP^106–126^, may be a novel mechanism of mitochondrial damage and disruption of homeostasis in prion diseases. SIRT1 participates in modulating mitochondrial morphology and function, and the SIRT1-dependent PGC-1α-TFAM signaling pathway plays a key role in the regulation of mitochondrial biogenesis in PrP^106–126^-treated N2a cells. Notably, RSV, a common natural polyphenol, activates mitochondrial biogenesis by triggering the SIRT1-dependent PGC-1α/TFAM signaling pathway to alleviate PrP^106–126^-induced mitochondrial dysfunction and cell apoptosis in N2a cells.

Recent studies have described multiple hallmarks of NDDs, including prion diseases. Altered energy homeostasis is one of these hallmarks [22]. Mitochondria are considered the “powerhouses” of the cell and are the primary energy source, providing ATP through oxidative phosphorylation to maintain neuronal homeostasis and function. To date, many studies have described extensive mitochondrial abnormalities in the neurons of NDD patients. Thus, neuronal mitochondrial dysfunction is considered an early and prominent feature of NDDs [19,54,55,56]. In this study and previous studies from our laboratory [16,17,18], mitochondrial morphological damage and dysfunction were evident in prion disease models, both in vitro and in vivo.

As highly metabolically active cells with high energy demands, neurons are particularly sensitive to changes in mitochondrial homeostasis. MQC can surveil and eliminate damaged mitochondria during aging or disease and restore mitochondrial homeostasis and maintain a healthy pool of mitochondria in neurons [20,27]. As a process that contributes to MQC, mitochondrial biogenesis regulates mitochondrial homeostasis in neurons and promotes neuron health by increasing and balancing the number of effective mitochondria in the cytoplasm [20,26,27,28]. Remarkably, we found that mitochondrial biogenesis was impaired in N2a cells following PrP^106–126^ exposure, as demonstrated by a decline in mtDNA copy number and decreased expression of mitochondrial-encoded (MTCO2 and MT-cytb) and nuclear genome-encoded mitochondrial proteins (SDHA and NDUFB8).

The decrease in mtDNA copy number and impairment of mitochondrial biogenesis observed in our prion disease model have been reported in aging and other NDDs. Some recent studies have observed that mtDNA copy numbers were reduced by 7–14% in AD compared to control participants [57], and higher mtDNA copy number levels reflect healthier mitochondria in elderly brains [58]. Moreover, mitochondrial biogenesis has been found to be defective in iPSC-derived dopaminergic neurons [59] and frontal cortex neurons [60] from PD patients, as well as in hippocampus and pyramidal neurons from AD patients [61].

We further explored the causes and specific regulatory mechanisms that underlie the impairment of mitochondrial biogenesis in prion diseases. Mitochondrial biogenesis is a complex and multistep process, and one essential step is the activation of transcription factors that regulate the genes critical for mitochondrial function. PGC-1α is the master regulator of mitochondrial biogenesis and can be activated by SIRT1; activated PGC-1α subsequently increases the transcription of nuclear target genes encoding the proteins involved in mitochondrial biogenesis, such as TFAM. TFAM, as a mitochondrial biogenesis effector downstream of PGC-1α, is the final effector of mitochondrial DNA transcription and replication [26,62]. We demonstrated that the SIRT1-PGC-1α-TFAM signaling pathway promotes mitochondrial biogenesis in the prion disease cell model. Our results are consistent with other studies in NDDs and other neurological injury models, which have also described the SIRT1-PGC-1α-TFAM signaling pathway as a major signaling pathway involved in the regulation of mitochondrial biogenesis [33,34,63,64]. Other pathways involved in the regulation of mitochondrial biogenesis have also been reported, such as the direct activation of PGC-1α by AMPK [65,66] or indirect activation of PGC-1α through phosphorylating FNIP1 [67] or modulating NAD+ metabolism and SIRT1 activity [68,69]. Additionally, PGC-1α promotes gene transcription by interacting with transcription factors such as estrogen-related receptor alpha (ERRα), peroxisome proliferator-activated receptor γ (PPARγ), and nuclear respiratory factor 1/2 (NRF1/2) [70]. Some studies have found that NRF1/2 is involved in PGC-1α-related mitochondrial biogenesis regulation [71,72,73]. Other pathways involved in the regulation of mitochondrial biogenesis need to be further explored in the context of prion diseases.

Our findings indicated that the pharmacological effects of SRT1720 and EX527 extend beyond merely enhancing the deacetylation activity of SIRT1. These compounds also modulate the protein expression levels of SIRT1 to a certain degree in the PrP^106–126^-induced prion disease model. The precise pharmacological mechanisms underlying these observations warrant further investigation. Furthermore, this study also identified that SIRT1 interference, overexpression, activation, and inhibition not only influenced the activity of PGC-1α but also impacted its expression. This effect may be mediated through SIRT1’s regulation of AMPK, subsequently affecting PGC-1α gene transcription [65,74,75]. The precise mechanisms underlying these interactions warrant further investigation, particularly in the context of prion diseases.

In addition, we observed that the expression and activity of SIRT1 decreased in PrP^106–126^-treated N2a cells, which inhibited the SIRT1-PGC-1α-TFAM signaling pathway and may lead to impaired mitochondrial biogenesis and mitochondrial dysfunction. Our results showed that the decrease in SIRT1 activity in PrP^106–126^-treated N2a cells is related to the observed reduction in NAD+ levels, which may be due to competitive inhibition by other NAD+-consuming enzymes, including poly(ADP-ribose) polymerases (PARPs), particularly PARP1 [76,77], cyclic ADP-ribose synthases (cADPRSs) [78], and SARM1 [40]. Importantly, the subsequent supplementation of NAD+ during PrP^106–126^ treatment restored SIRT1 deacetylase activity to that observed under normal conditions in N2a cells. In this study, we successfully restored SIRT1 activity through the direct supplementation of NAD+. Considering that NAD+ cannot simply diffuse through cell membranes, this observed effect is likely attributable to the activity of cell membrane-bound glycohydrolases, such as CD73, which facilitate the conversion of extracellular NAD+ into NMN or NR, thereby enabling their transport across the membrane and into the intracellular environment [79]. However, as a multifunctional NAD+-dependent deacetylase, SIRT1 attenuates the damage and dysfunction of mitochondria through regulating not only mitochondrial biogenesis but also another MQC pathways like mitophagy [80,81] and mitochondrial dynamics [82,83,84]. Previous work carried out in our lab showed that PINK1-parkin-mediated neuronal mitophagy is defective in PrP^106–126^-incubated N2a cells [16]. Whether this finding was related to the decreased expression and activity of SIRT1 remained unclear. Thus, these processes will be the subject of our subsequent studies.

RSV is a natural multitarget polyphenol compound with pleiotropic activity [85]. A previous study showed that RSV treatment significantly increased both SIRT1 protein expression and SIRT1 activity and inhibited PrP^106–126^-induced cell death in neuroblastoma cells [37]. What we found on the effect of RSV on PrP^106–126^-treated neuroblastoma cell survival is consistent with previous studies. Beyond this, we further observed that RSV protects N2a cells from PrP^106–126^-induced mitochondrial dysfunction and cell apoptosis by activating mitochondrial biogenesis regulated by the SIRT1-dependent PGC-1α/TFAM signaling pathway. Interestingly, we determined that the protective effect of RSV against PrP^106–126^-induced mitochondrial dysfunction and cell apoptosis was reversed by SIRT1 knockdown, suggesting that the beneficial effects of RSV on N2a cell MQC require SIRT1. Similar results were obtained in other studies demonstrating the beneficial effects of RSV in several in vitro and in vivo models of NDDs and aging via modulation of the SIRT1-PGC-1α pathway [86,87,88].

In summary, impaired mitochondrial biogenesis regulated by the SIRT1-PGC-1α-TFAM pathway potentially contributes to mitochondrial damage and plays a critical role in the pathogenesis of prion diseases. The significant downregulation of SIRT1 in PrP^106–126^-treated N2a cells and the ability of SIRT1 to positively regulate MQC make it a potential target for therapeutic intervention in prion diseases. RSV, as a widely studied compound with demonstrated neuroprotective properties, alleviates mitochondrial morphological damage and dysfunction and inhibits cell apoptosis by targeting mitochondrial biogenesis via SIRT1 in PrP^106–126^-incubated N2a cells. These findings provide novel insights for a better understanding of the mitochondrial-related pathogenesis of prion diseases and supports SIRT1 as a potential target for therapeutic intervention of prion disease.

However, our study also has some limitations that need to be addressed in future research. It is important to acknowledge that the SIRT1 activity assessed in this study does not represent the natural SIRT1 activity within each sample group. Nevertheless, a constant concentration of NAD+ provided in the assay kit was utilized to measure the SIRT1 concentration capable of exerting deacetylase activity across the groups, thereby reflecting the relative differences in SIRT1 deacetylation activity among the groups to a certain extent. Furthermore, the selection of PrP^106–126^ concentration in this study was informed by prior research on mitochondrial damage in prion disease [17,18,40]. Based on the findings presented in Section 2.1 of this study, we ultimately chose a concentration of 150 µM PrP^106–126^ to treat N2a cells for a duration of 24 h. This specific condition was determined to be optimal for the investigation of the SIRT1-mediated mitochondrial biogenesis pathway in prion diseases. While alternative concentrations of PrP^106–126^ and varying incubation times may be applicable in other prion disease-related research, they fall outside the primary scope of this study and, therefore, were not incorporated into subsequent experiments following the initial result. Though N2a cells are widely used in the study of PrP^106–126^ toxicity, they may not completely recapitulate the biological characteristics of neurons in vivo. The regulation of SIRT1 on mitochondrial biogenesis in prion diseases will need to be further demonstrated in primary neurons and in vivo.

## 4. Materials and Methods

### 4.1. Cell Culture and Treatments

Mouse neuroblastoma (N2a) cells were obtained from the Cell Resource Center (IBMS, CAMS/PUMC, Beijing, China) and were cultured in DMEM (HUANKE, Beijing, China) with 10% fetal bovine serum (FBS) (Gibco, Grand Island, NY, USA) and 1% penicillin/streptomycin at 37 °C in a humidified incubator with 5% CO_2_. N2a cells were confirmed to be mycoplasma-free by PCR.

The PrP peptide PrP^106–126^ (sequence: KTNMKHMAGAAAAGAVVGGLG, 98% purity) was synthesized by YaMei Peptides Bio-Tech (Beijing, China), and the scramble PrP^106–126^ peptide (sequence: MEVGWYRSPFSRVVHLYRNGK, 95% purity), which was used as a control, was synthesized by Sangon Bio-Tech (Shanghai, China). Both peptides were dissolved in phosphate-buffered saline (PBS) at a concentration of 1 mM and shaken for 24 h at 4 °C. Except for the results specified in Section 2.1, the experimental conditions in the rest of the results were N2a cells treated with 150μ M PrP^106–126^ for 24 h.

β-Nicotinamide adenine dinucleotide (β-NAD; Sigma-Aldrich, St. Louis, MO, USA) was dissolved in PBS to a storage concentration of 10 mM and stored protected from light at −20 °C. SRT1720 and EX527 were purchased from Selleckchem (Houston, TX, USA), and resveratrol was purchased from ApexBio (Houston, TX, USA). NAD, SRT1720, EX527, and resveratrol were used at working concentrations of 25 μM, 10 μM, 10 μM, and 10 μM, respectively. Each reagent was added to the cell culture medium 1 h prior to the administration of the PrP^106–126^ treatment.

### 4.2. Small Interfering RNAs, Plasmids, and Transfections

*SIRT1* small interfering RNA (siRNA; sense: 5′-CCGUCUCUGUGUCACAAAUTT-3′; antisense: 5′-AUUUGUGACACAGAGACGGTT-3′) was obtained from Synbio Technologies (Suzhou, China). *TFAM* siRNA (sense: 5′-GCAUGGGUAGCUAUCCAAATT-3′; antisense: 5′-UUUGCAUAGCUACCCAUGCTT-3′) and *PGC-1α* siRNA (sense: 5′-GAAGUGGUGUAGCGACCAATT-3′; antisense: 5′-UUGGUCGCUACACCACUUCTT-3′) were obtained from Tsingke Biotechnology (Beijing, China). The pcDNA3.1(+)-SIRT1, pcDNA3.1(+)-TFAM, and pcDNA3.1(+)-PGC-1*α* plasmids were obtained from Synbio Technologies. The DsRed-Mito plasmids were obtained from Clontech (Mountain View, CA, USA).

The siRNAs were transfected into N2a cells using antibiotic-free DMEM, supplemented with 1.5 μL of Lipofectamine 3000, 50 μL of Opti-MEM, and 25 pmol of siRNA per 500 μL of cell culture medium, for a duration of 8–10 h. Plasmids were transfected into N2a cells using 0.75 μL Lipofectamine 3000, 50 μL Opti-MEM, 1 μL P3000 reagent, and 500 ng plasmids per 500 μL of antibiotic-free DMEM for 8–10 h. The transfection medium was then removed and replaced with fresh culture medium. After approximately 48 h from the point of transfection, experimental treatments on the cells were initiated. All transfection reagents were purchased from Invitrogen (Carlsbad, CA, USA).

### 4.3. Western Blot Assays

Western blotting was performed using a previously published method [36]. N2a cells were seeded at a density of 1.2 × 10^5^ cells/well in 24-well plates and subsequently harvested after the experimental processes using a lysis buffer supplemented with 1% protease inhibitor PMSF (Solarbio, Beijing, China, catalog no. R0020). A volume of 50 μL of lysis solution is required for each well in a 24-well plate. The lysed cells were collected and cultured with lysis buffer in Eppendorf tubes, followed by centrifugation at 12,000× *g* for 10 min at 4 °C. The supernatants were subsequently transferred to new tubes. The samples were subjected to Western blot analysis following denaturation with a 5× SDS-PAGE loading buffer (Solarbio, Beijing, China, catalog no. P1040) through boiling. The samples were subjected to electrophoresis on a 10% SDS-PAGE gel initially at 60 V for 30 min, followed by 120 V for 70 min. Subsequently, the proteins were transferred onto a polyvinylidene difluoride (PVDF) membrane (Millipore, Billerica, MA, USA catalog no. ISEQ00010) at 100 V for 120 min. The membrane was then blocked with 5% skimmed milk (Solarbio, Beijing, China catalog no. D8340) at 37 °C for 1 h. The following primary antibodies and dilutions were used for detection: SIRT1 (1:2000, Proteintech, Wuhan, China, catalog no. 60303-1-lg), TFAM (1:1000, Abcam, Cambridge, MA, USA, catalog no. ab252432), PGC-1α (1:5000, Proteintech, Wuhan, China, catalog no. 66369-1-Ig), MTCO2 (1:2000, Proteintech, Wuhan, China, catalog no. 55070-1-AP), MT-cytb (1:1000, Proteintech, Wuhan, China, catalog no. 55090-1-AP), SDHA (1:1000, Abcam, Cambridge, MA, USA, catalog no. ab137040), NDUFB8(1:1000, Abcam, Cambridge, MA, USA, catalog no. ab192878), GAPDH (1:5000, Proteintech, Wuhan, China, catalog no. 60004-1-Ig), β-tubulin (1:5000, Proteintech, Wuhan, China, catalog no. 10094-1-AP), cleaved caspase-3 (1:1000, Cell Signaling Technology, Danvers, MA, USA, catalog no. 9664), and caspase-9 (1:1000, Proteintech, Wuhan, China, catalog no. 10380-1-AP). Following the washing of each membrane with Tris-buffered saline containing 0.1% Tween 20, the membranes were subsequently incubated with the appropriate secondary antibodies. The following secondary antibodies were used: HRP-conjugated Affinipure Goat Anti-Rabbit IgG (H+L) (1:10,000, Proteintech, Wuhan, China, catalog no. SA00001-2) and HRP-conjugated Affinipure Goat Anti-Mouse IgG (H+L) (1:10,000, Proteintech, Wuhan, China, catalog no. SA00001-1). Blots were developed using ECL Western detection reagents (Bio-Rad, Hercules, CA, USA) and visualized using a chemiluminescent imaging system (Tanon Science and Technology, Shanghai, China). Images were analyzed using ImageJ software version 1.52a (National Institutes of Health, Bethesda, MD, USA).

### 4.4. Measurement of SIRT1 Deacetylase Activity

SIRT1 deacetylase activity was measured using a SIRT1 activity assay kit (Abcam, ab156065) according to the manufacturer’s protocol. Briefly, N2a cells were seeded at a density of 2 × 10^6^ cells/100mm cell culture dish, and after experimental treatments, the proteins were extracted, and the fluorescence intensities were measured at Ex/Em = 350/460 nm with a Spark multimode microplate reader (TECAN, Männedorf, Switzerland). The experimental values were normalized to those measured for the control. In this experimental kit, a fluorophore and a quencher are conjugated to the amino and carboxyl termini of a substrate peptide, respectively. Prior to the deacetylase reaction, fluorescence emission is inhibited. Upon deacetylation by SIRT1, the substrate peptide undergoes cleavage by a concurrently added protease, resulting in the separation of the quencher from the fluorophore and subsequent fluorescence emission. The activity of the deacetylase enzyme is quantified by measuring the intensity of the emitted fluorescence.

### 4.5. Measurement of NAD+ Levels

N2a cells were seeded at a density of 2 × 10^5^ cells/well in 12-well plates. The NAD/NADH assay kit with WST-8 (Beyotime, Shanghai, China, catalog no. S0175) was used to determine the cellular NAD+ according to the manufacturer’s protocol. Formed formazan was quantified by measuring the absorbance of each well at 450 nm using a microplate reader (Thermo Fisher, Waltham, MA, USA). All values were normalized to those measured for the control.

### 4.6. Detection of ATP

An enhanced ATP Assay Kit (Beyotime, Shanghai, China, catalog no. S0027) was used to determine the ATP levels according to the manufacturer’s instructions. N2a cells were seeded in 24-well cell culture plates, lysed with the lysis buffer provided in the kit after experimental treatments, and then incubated in white 96-well plates with 100 μL detection reagent, and 20 μL of cell samples was added into each well. Luminescence was measured using a Spark multimode microplate reader (TECAN, Männedorf, Switzerland). Experimental values were normalized to those measured for the control.

### 4.7. Measurement of the Mitochondrial Membrane Potential

A MMP assay kit with JC-1 (Beyotime, Shanghai, China, catalog no. C2006) was used to detect MMP as described by the manufacturer. The N2a cells were seeded at a density of 2 × 10^5^ cells/well in 12-well plates. Following the experimental treatments, the N2a cells were collected in a tube, washed three times with PBS, and were then incubated with the JC-1 probe for 30 min at 37 °C. The cells were washed three times with the kit wash buffer and were then examined using FACS Calibur (BD Biosciences, San Jose, CA, USA).

### 4.8. Immunofluorescence Microscopy

N2a cells were seeded in 24-well plates with cover slips and were treated with the indicated siRNAs and plasmids, along with the DsRed-Mito plasmid, for 48 h. After experimental treatment, the cells were gently washed with PBS and incubated with Immunol Staining Fix Solution (Beyotime, Shanghai, China, catalog no. P0098) at 37 °C for 30 min, followed by Immunol Permeabilization Buffer (Beyotime, Shanghai, China, catalog no. P0095) at room temperature for 5 min. Following the washing with PBS buffer, DAPI (Solarbio, Beijing, China, catalog no. C0065) was used to stain the nucleus at 37 °C for 5 min, and the slides were sealed with anti-fluorescence quenching sealing tablets (Bioworld Technology, Bloomington, MN, USA). Fluorescence images of the mitochondria were acquired using an A1 confocal microscope (Nikon, Tokyo, Japan), and the mitochondrial lengths were measured using Image J 1.52a (National Institutes of Health, Bethesda, MD, USA).

### 4.9. Reverse Transcription-Quantitative Real-Time PCR (RT-qPCR)

The N2a cells were seeded at a density of 5 × 10^5^ cells/well in 6-well plates. Total RNA from the N2a cells was extracted using an EASYspin Plus kit (Aidlab, Beijing, China) according to the manufacturer’s instructions. Reverse transcription was performed using the HiScript^®^ III All-in-One RT SuperMix Perfect for qPCR (Vazyme, Nanjing, China, catalog no. R333). The resulting cDNA was quantified by spectrophotometry (Thermo NanoDrop 2000, Wilmington, DE, USA) and stored at −80 °C until use. Quantitative PCR was performed using Taq Pro Universal SYBR qPCR Master Mix (Vazyme, Nanjing, China, catalog no. Q712) on an ABI StepOnePlus Real-time fluorescence quantitative PCR instrument (Thermo Fisher Scientific, Waltham, MA, USA). The primer sequences are listed in Table S1 and were synthesized by Tsingke Biotechnology (Beijing, China). All expression values were normalized to the control, GAPDH, and relative gene expression levels calculated using the 2^−ΔΔCt^ method.

### 4.10. Assessment of the Mitochondrial DNA (mtDNA) Copy Numbers

The N2a cells were seeded at a density of 5 × 10^5^ cells/well in 6-well plates. Total DNA was extracted from N2a cells using the EasyPure Genomic DNA kit (Transgen, Beijing, China, catalog no. EE101) and was quantified by spectrophotometry (Thermo NanoDrop 2000, Wilmington, DE, USA). The mtDNA and nuclear DNA (nDNA) were assessed by RT-qPCR using the primers described in Table S1. The mtDNA copy number was determined using the ratio of mtDNA to nDNA, which were calculated using the 2^−ΔΔCt^ method and represented as the fold change relative to the control.

### 4.11. Cell Viability Assays

The Cell Counting Kit-8 assay kit (CCK-8 kit, Beyotime, Shanghai, China, catalog no. C0038) was used to evaluate the N2a cell viability following the experimental treatments. The absorbance of each sample at 450 nm was determined using a microplate reader (Thermo Fisher, Waltham, MA, USA). The experimental values were normalized to those measured for the control.

### 4.12. TUNEL Assays

N2a cell apoptosis was evaluated by TUNEL assays using the One Step TUNEL Apoptosis Assay Kit (Beyotime, Shanghai, China,catalog no. C1086) according to the manufacturer’s instructions. The cells were visualized using an A1 confocal microscope (Nikon, Tokyo, Japan).

### 4.13. Statistical Analyses

All assays were performed at least three times, and replicates were represented by individual data points in each graph. Data are expressed as the mean ± SEM. Parametric data were analyzed using ordinary one-way or two-way analysis of variance (ANOVA) with Tukey’s or Sidak’s multiple comparisons test. Analyses were performed using Prism 9.0 software (GraphPad Software, La Jolla, CA, USA). *p*-values < 0.05 were considered statistically significant.

## Data Availability

Most of the data generated or analyzed during this study are included in this published article and its supplementary information files. Additional datasets generated are available from the corresponding author upon reasonable request.

## Supplementary Materials

The following supporting information can be downloaded at https://www.mdpi.com/article/10.3390/ijms25179707/s1.
