# Supplementary material for: SIRT1 Regulates Mitochondrial Damage in N2a Cells Treated with the Prion Protein Fragment 106–126 via PGC-1α-TFAM-Mediated Mitochondrial Biogenesis
Source: Int J Mol Sci. 2024 Sep 7;25(17):9707. doi: 10.3390/ijms25179707 (PMC11395710; doi:10.3390/ijms25179707)
Supplement: Supplementary file 1 [file ijms-25-09707-s001.zip › ijms-3141991-supplementary.pdf]

Supplementary Informations

Supplementary Tables

Table S1. Sequences of primers used in RT−qPCR for measurement of mRNAs and mtDNA.

Gene name		Sequence (5' − 3')
12sRNA (mtDNA)	F	ACCGCGGTCATACGATTAAC
	R	CCCAGTTTGGGTCTTAGCTG
18sRNA (nDNA)	F	CATTCGAACGTCTGCCCTATC
	R	CCTGCTGCCTTCCTTGGA
PGC-1α	F	GAATCAAGCCACTACAGACACCG
	R	CATCCCTCTTGAGCCTTTCGTG
TFAM	F	AAGGATGATTCGGCTCAGG
	R	GGCTTTGAGACCTAACTGG
MTCO2	F	GCCGACTAAATCAAGCAACA
	R	CAATGGGCATAAAGCTATGG
MT−cytb	F	CATTTATTATCGCGGCCCTA
	R	TGTTGGGTTGTTTGATCCTG
GAPDH	F	CCGAGAATGGGAAGCTTGTC
	R	TTCTCGTGGTTCACACCCATC

Supplementary Figure

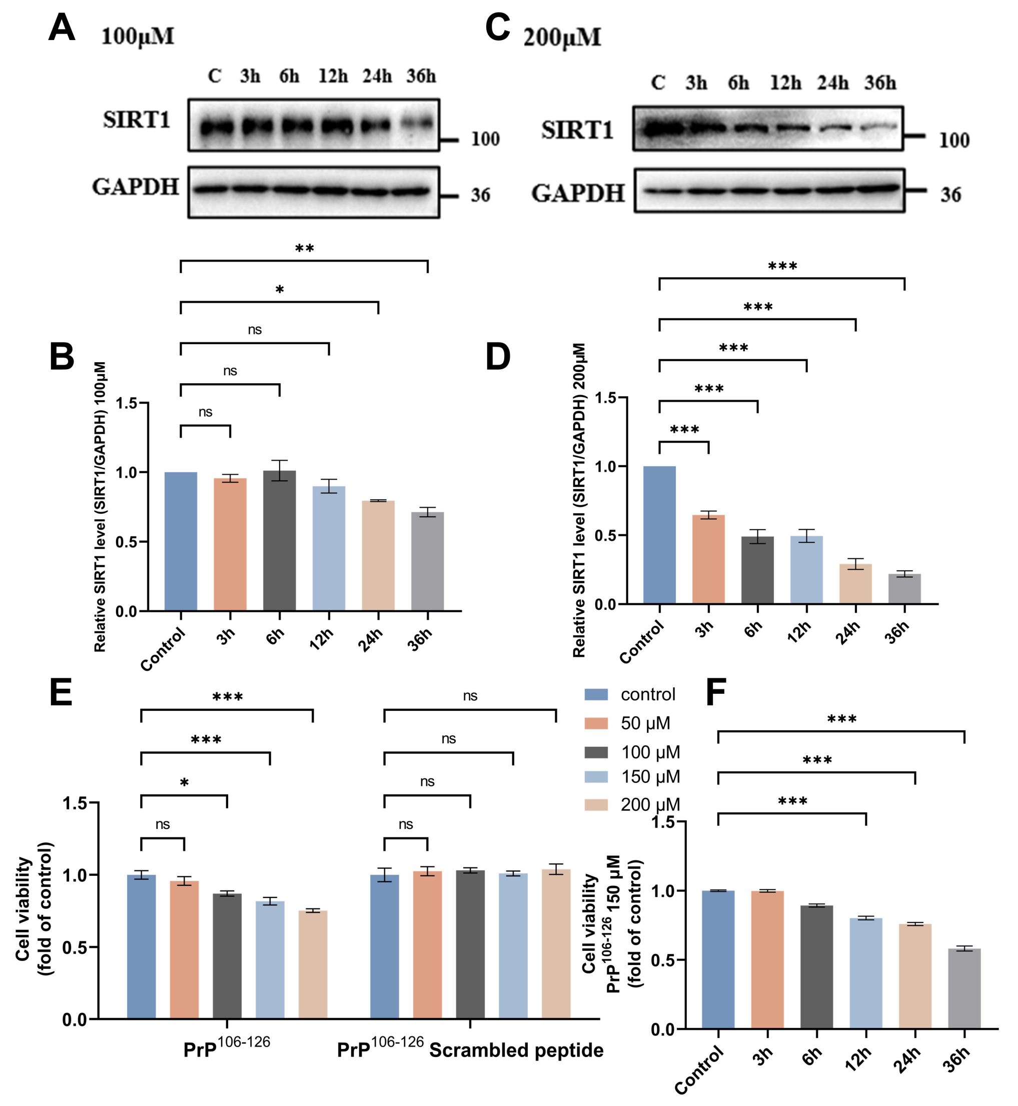

Figure S1. SIRT1 expression is downregulated in PrP$^{106-126}$–exposed N2a cells.

(A) Western blots of SIRT1 from N2a cells treated with 100 μM PrP$^{106-126}$.

(B) Quantitation of SIRT1 expression shown in (A).

(C) Western blots of SIRT1 from N2a cells treated with 200 μM PrP$^{106-126}$.

(D) Quantitation of SIRT1 expression shown in (C).

(E) Cell viability was assayed using the CCK−8 kit in N2a cells with different concentrations of PrP$^{106-126}$ or PrP$^{106-126}$ scrambled peptide for 24h.

(F) Cell viability was assayed using the CCK−8 kit in N2a cells treated with 150 μM PrP$^{106-126}$ at different time points.

Data are expressed as the mean ± SEM. Statistical significance was analysed via

ordinary one−way ANOVA with Tukey's multiple comparisons test for (B,D) or two−way ANOVA with Sidak's multiple comparisons test for (E). n=at least 3 biologically independent treatments/transfections of cells for each. ns, not significant; *$P < 0.05$; **$P < 0.01$; ***$P < 0.001$.
